# Validation and clinical correlation of triplex CD3, CD8 and FOXP3 IHC of tumor-infiltrating lymphocytes in follicular lymphoma

**DOI:** 10.1186/2051-1426-2-S3-P261

**Published:** 2014-11-06

**Authors:** James Mansfield, Christian Slater, Richard Byers

**Affiliations:** 1 PerkinElmer, Hopkinton, MA, USA; 2 University of Manchester, Manchester, UK

## 

It is clear that immune cells play many sometimes conflicting roles in the tumor microenvironment and it would be extremely useful to be able to visualize the distributions of multiple phenotyped immune cells in-situ in solid tumors. However, obtaining phenotypic information about the various cells that play these roles in and around the tumor has been a challenge. Existing methods can either deliver phenotypic information on homogenous samples (e.g., flow cytometry) or morphologic information on single immunomarkers (standard IHC). These limitations can be largely overcome through a multiplexed staining, imaging and analysis methodology using standard clinical FFPE tissue sections. Although multiplex methods have been shown to be particularly useful not much has been presented on the validation of such methods. We present here a validation of the method for CD3, CD8 and FOXP3 in tissue microarray containing triplex follicular lymphoma cores from 40 subjects [24 male, 16 female, age 35 to 75 years at diagnosis, median 55 years, 2- 171 months follow-up]. This involves a sequential multi-marker labeling for 3 antigens and a counterstain; automated multispectral imaging to separate chromogens; and an automated analysis that can quantitate the per-cell marker expression, determine the cellular phenotype, count these cells separately in the tumor compartment and in the stroma and provide high-resolution images of their distributions. The IHC for each marker was optimized in as single-stain IHC and then those specifications used as a part of the triplex stain. The samples were scored using an automated scoring methodology and the results from the triplexed method are compared to analyses done on singly stained sections with excellent correlation (R greater than 0.9 in all cases), showing that multiplexed staining methods can replicate standard IHC methods while maintaining the inter-distribution and visualization of the markers in a single section.

